# Ethnoterritorial features of paranoid schizophrenia with comorbid dependence on synthetic cannabinoids: Description of cases

**DOI:** 10.1192/j.eurpsy.2025.232

**Published:** 2025-08-26

**Authors:** N. Bokhan, G. Selivanov

**Affiliations:** 1 Mental Health Research Institute, Tomsk; 2 Psychiatric Hospital of St. Nicholas the Chudotvorets, St. Petersburg, Russian Federation

## Abstract

**Abstract: Introduction:**

it is indisputable that against the backdrop of the popularity of various addictions among patients with schizophrenia, a joint study of the clinical and pathomorphological deformation of two nosologically phenomena of paranoid schizophrenia and, depending on modern drugs that are gaining popularity, synthetic cannabinoids in various ethnoterritorial groups is relevant.

**Objectives:**

to study the ethnoterritorial features of paranoid schizophrenia, comorbid with the abuse of synthetic cannabinoids, clinical dynamics, behaviour, and adaptation.

**Methods:**

follow-up, clinical-psychopathological methods (PANS, SANS, CGI, MMPI, CGI, STAI, LSI, TPA, ICD-10), statistical (Python 3.11.0).

**The results:**

of the examination of 193 patients (aged from 18 to 35 years): 142 – patients with paranoid schizophrenia dependent on synthetic cannabinoids F20.xx+F12.2xx and 51 – F20.xx without drug addiction. The study took place from 2018 to 2024 in the database of psychiatric institutions in Russia - Tomsk region, St. Petersburg, Noyabrsk and Nizhnevartovsk.

**Conclusions:**

The leading position among patients with schizophrenia who use synthetic cannabinoids in the temperate continental climate zone of Russia was occupied by such ethnic groups as the hierarchy: Russians, Tatars; Uzbeks; Germans; Azerbaijanis, and Armenians.

The phenomenon of abuse of synthetic cannabinoids leads to the development of diseases. Persistent exogenous visual and delirious disorders are included in the complex of symptoms of exacerbation of schizophrenia; A new symptom of pseudohallucinoids appears - thought disorders of an associative (fantasy) disease that arose against the background of long-term exogenous (toxic) effects of the drug on the subject type, usually against the background of a primary endogenous schizophrenic process

**Disclosure of Interest:**

None Declared

